# The art of limitations

**DOI:** 10.1007/s40037-015-0181-0

**Published:** 2015-05-30

**Authors:** Lorelei Lingard

**Affiliations:** Department of Medicine, Western University, Schulich School of Medicine & Dentistry, Health Sciences Addition, Rm. 112, N6A 5C1 London, ON Canada

In the writer’s craft section we offer simple tips to improve your writing in one of three areas: Energy, Clarity and Persuasiveness. Each entry focuses on a key writing feature or strategy, illustrates how it commonly goes wrong, teaches the grammatical underpinnings necessary to understand it and offers suggestions to wield it effectively. We encourage readers to share comments on or suggestions for this section on Twitter, using the hashtag: #how’syourwriting?

The Limitations section of a research paper is critically important but rarely effectively used. Writers tend to take one of three approaches: The Confessional, The Dismissal or The Reflection. There are understandable rhetorical motivations underpinning each approach, but only one fulfils the purpose of a Limitations section.

In The Confessional, the writer asks readers to forgive the flaws in study design. For instance: *Data collection occurred in a single institutional setting due to limited study resources. Faculty who agreed to respond to the survey may represent a biased sample*.

In The Dismissal, the writer acknowledges concerns only to dismiss their importance:

Observational research can produce Hawthorne effect, in which participants alter their naturalistic behaviour due to the observer’s presence. However, we are confident that the practices described in our study represent a robust range of possible strategies that faculty providing clinical feedback to trainees might realistically employ.

Writers taking this approach are recognizable by this admit/dismiss pattern. Yes, the sample size is small, but we are confident it includes the major points of view. Okay, the statistical tests were a fishing expedition, but our p value was significant.

In The Reflection, the writer lays out the aspects of the research design that create uncertainty about the knowledge contribution, paying attention to the nature of the uncertainty and its implications [1]. For example:

Our decision to sample academic paediatricians may explain the predominance of research as a feature of innovation in our results, due to the high value placed on research in academic settings. Future work could explore the relevance of our model outside the academic setting by interviewing community paediatricians regarding what constitutes ‘innovation’ in their everyday practice. While the use of an insider to conduct study interviews was effective in creating in depth discussions, it might also explain the limited discrepant opinions about the appropriateness or safety of innovations in paediatric practice. Our use of adaptive expertise as the theoretical underpinning for the research draws our attention to cognitive aspects of innovation, but diminishes other relevant aspects, such as organizational or cultural.

This reflective stance applies to technical details, such as the particular interview procedure, as well as to more conceptual issues such as the chosen theoretical framework. In both instances, the focus is on how the research approach creates uncertainties or blind spots in the knowledge that is produced. Ideally, writers should signal the impact of these uncertainties or blind spots on the relevance of the work—that is, on how readers might appropriately apply its contributions.

These three approaches to the Limitations section are responses to the social context of peer review. The Confessional enacts the assumption that the writer will be rewarded for recognizing their study flaws—better to point them out yourself than to have reviewers think you aren’t aware of them. The Dismissal likely arises from the experience that journal reviewers use identified limitations as a basis for rejecting manuscript submissions, so the writer needs to convincingly argue that none of the limitations are fatal. The Reflection is the only approach that gets to the heart of what a limitations section ought to accomplish: a considered argument about the sources of uncertainty in the research and what they mean for how a particular knowledge contribution should be taken up by others. This kind of Limitations section takes more space, and it may be vulnerable to reviewers looking for reasons to reject. But it is the type of Limitations discussion that we should be producing, as it is the only one that authentically supports the cumulative progression of knowledge in our scientific community.
